# Radiation Exposure from CT Examinations in Japan

**DOI:** 10.1186/1471-2342-10-24

**Published:** 2010-11-02

**Authors:** Yoshito Tsushima, Ayako Taketomi-Takahashi, Hiroyuki Takei, Hidenori Otake, Keigo Endo

**Affiliations:** 1 Department of Diagnostic Radiology and Nuclear Medicine, Gunma University Hospital 3-39-22 Showa-machi, Maebashi, Gunma, Japan

## Abstract

**Background:**

Computed tomography (CT) is the largest source of medical radiation exposure to the general population, and is considered a potential source of increased cancer risk. The aim of this study was to assess the current situation of CT use in Japan, and to investigate variations in radiation exposure in CT studies among institutions and scanners.

**Methods:**

Data-sheets were sent to all 126 hospitals and randomly selected 14 (15%) of 94 clinics in Gunma prefecture which had CT scanner(s). Data for patients undergoing CT during a single month (June 2008) were obtained, along with CT scan protocols for each institution surveyed. Age and sex specific patterns of CT examination, the variation in radiation exposure from CT examinations, and factors which were responsible for the variation in radiation exposure were determined.

**Results:**

An estimated 235.4 patients per 1,000 population undergo CT examinations each year, and 50% of the patients were scanned in two or more anatomical locations in one CT session. There was a large variation in effective dose among hospitals surveyed, particularly in lower abdominal CT (range, 2.6-19.0 mSv). CT examinations of the chest and upper abdomen contributed to approximately 73.2% of the collective dose from all CT examinations. It was estimated that in Japan, approximately 29.9 million patients undergo CT annually, and the estimated annual collective effective dose in Japan was 277.4 *10^3 ^Sv person. The annual effective dose per capita for Japan was estimated to be 2.20 mSv.

**Conclusions:**

There was a very large variation in radiation exposure from CT among institutions surveyed. CT examinations of the chest and upper abdomen were the predominant contributors to the collective dose.

## Background

Since computed tomography (CT) was introduced to medical practice, this diagnostic X-ray technique has provided great benefits for health care. In most circumstances, the risk to an individual patient of developing a malignant tumour because of CT is low and acceptable compared to the substantial benefit, although there is a large uncertainty in risk estimates at these dose levels. However, the large number of people exposed means that even small individual risks could translate into a considerable number of cancer deaths [1,2]. In the UK (2005-2006), approximately 60% of the total radiology collective effective dose was from CT [3]. In Germany (2000-2005), the contribution of CT to the collective effective dose for cancer patients from all X-ray procedures was approximately 82% [4]. In the USA, about 67% of the collective effective dose in diagnostic radiology was due to CT scanning [5]. These data indicated that CT represents more than half the radiation exposure from diagnostic imaging. In Japan, there is unfortunately no such reliable data regarding radiation exposure from radiological imaging, but the situation is likely not very different.

We surveyed all individual patients undergoing CT examinations during a period of one month in a single prefecture (state) in Japan. Age and sex distribution, anatomical locations scanned and radiation exposure of the patients receiving scans were noted.

## Methods

We collected data concerning individual patients undergoing CT in Gunma prefecture during a single month (June 2008), along with the standard CT scan protocols of each scanner used in the respective institutions. For each individual patient, organ doses were calculated and the effective doses were obtained (using the standard CT scanning protocol of each institute). From these data, the current situation of CT examinations in Japan, with particular attention paid to radiation exposure, was estimated. The epidemiological research ethics committee of Gunma University Faculty of Medicine approved this survey, and did not require any informed consent from each patient, since all the data used were anonymous and the data collection did not affect patient management in any way.

### • Collecting the data of individual patients undergoing CT and standard CT scanning protocols

**Figure 1:**
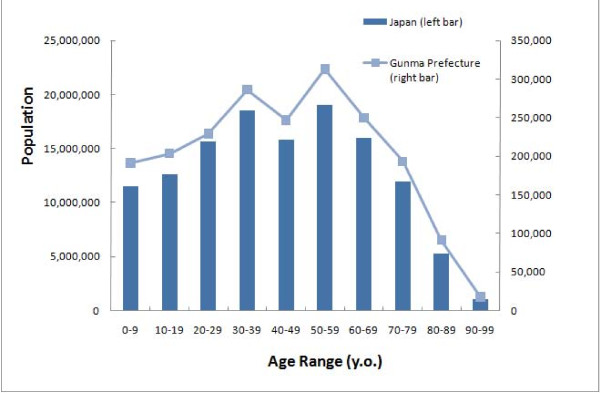
**Age distribution of the population of Japan and Gunma prefecture**. The distributions are very similar.

A questionnaire was mailed to the chief radiologic technologist in all 126 hospitals and randomly selected 14 (15%) of 94 clinics which had CT scanner(s) in Gunma prefecture (state). Gunma prefecture is located approximately 100 km north of Tokyo, and its population is two million. The age distribution in Gunma prefecture is very similar to that of Japan as a whole (Figure 1) [6], and we suspect that the medical environment in Gunma prefecture probably reflects that of the Japanese national average (Table 1) [7-11]. The chief technologist in each hospital was asked to make a list of all patients who underwent CT during a period of one month (June 2008). The data of each *patient *(CT session) consisted of the patient's age and sex, anatomical location of the CT scan and number of scans. When a patient returned for a second or more CT session on a different day, the sessions were counted as two different patients each undergoing a single CT session. The anatomical locations were divided into head, face, neck, chest, upper abdomen, lower abdomen (pelvis) and other.

**Table 1:** Medical environment of Gunma prefecture.

Variables	Gunma prefecture	National average	Year of the data (references)
**Population density (/km^2^)**	318	340	2006
**Annual income per capita (Japanese yen)**	2,828,000	2,978,000	2004 (21)
**Number of hospitals (/100,000 population)**	6.9	6.9	2007 (19)
**Number of beds (/100,000 population)**	1263	1268	2007 (20)
**Number of physicians (/100,000 population)**	208.6	217.5	2006 (17)
**Annual medical cost per capita (Japanese yen)**	343,000	386,000	2005 (18)

The annual medical cost per population in Gunma prefecture was about 10% lower compared to the national average, but other variables are almost average.

In many patients, more than two anatomical locations were scanned, and sometimes more than two scans were performed on one anatomical location in a single CT session. Therefore, we defined *the number of examinations *as the number of anatomical locations scanned, and *the number of scans *as the total number of scans for each anatomical location. For example, if a patient underwent unenhanced CT of the upper abdomen and enhanced CT of the upper and lower abdomen in a single CT session, the number of CT examinations was two, and the number of scans of the upper abdomen was two and that of the lower abdomen was one.

From the data obtained, the mean numbers of the hospitals or clinics were calculated, and were multiplied by the number of CT scanners to estimate the total number of patients, examinations, and scans. These numbers multiplied by 12 were estimated as being the annual number of each in Gunma prefecture. The population of Japan is approximately 127 million and that of Gunma prefecture is two million, so these annual numbers for Gunma prefecture were multiplied by 127/2 to estimate the annual numbers for those in Japan.

Inquiries were also made on the model of the CT scanner and standard scanning protocols for each anatomical location (tube voltage [kV], tube current [mA], rotation time [sec], collimation [mm], and pitch). Use of an automatic exposure control (AEC) system was also noted, if applicable.

### • Calculation of the organ doses for each patient

We employed ImPACT CT Patient Dosimetry Calculator version 0.99x http://www.impactscan.org/. This software, which is constructed using a Microsoft Excel spreadsheet, is a tool for calculating patient organ and effective doses from CT. It makes use of the NRPB Monte Carlo dose data sets produced in NRPB-SR250 [12], which provides normalized organ dose data for irradiation of a mathematical phantom by a range of CT scanners.

For each patient, organ doses (mSv) were calculated using this software. The z-axis (axial) extent of the scan for each type of CT examination (anatomical location) were fixed as follows: 14 cm for head, 10 cm for face, 10.5 cm for neck, 25 cm of chest, 20 cm for upper abdomen, and 25 cm for lower abdomen. Data for most modern CT scanners of major vendors were included in this software. If data from a CT scanner used in a hospital or clinic in this study was not found in the data included in this software, available data from the most similar CT scanner was used.

The phantom used to produce the Monte Carlo data sets in NRPB-SR250 [12] is based on a mathematical representation of an average adult, but does not address the issue of dose to paediatric patients. Therefore, in this study, we calculated organ doses only for the patients older than 20 years of age.

### • Estimations of effective dose for one scan or examination and annual collective dose in Japan

Estimated effective doses (mSv) per scan or examination for each anatomical location were obtained by using the same software. From the values obtained, collective doses during the single month in Gunma prefecture were estimated, then, multiplied by 12 to estimate the annual collective doses. The annual collective doses in Japan attributable to CT were estimated by multiplying this value by 127/2. The annual effective dose per capita was also estimated.

To find which factors may contribute to the estimated effective doses, these were correlated with hospital size (number of in-patient beds), the number of patients undergoing CT in one month, and the row number of the CT detector by using a linear regression analyses.

## Results

### • Data collection (Table 2)

**Table 2:** Number of CT scanners in Gunma prefecture and data collection.

	Number of CT scanners			Estimated annual number of patients		
	
	Total	Data obtained (%)				
					
		Complete data	Scanning protocol & Number of patients *	Mean	Range	Estimated total number
**Hospitals**	132	63	61	2,571	36 - 23,616	339,393
**Clinics**	94 **	6	0	1,406	288 - 2,664	132,164

**Total**	226	69	61			471,557

* No individual patients' data were provided.

** Data requests sent to 14 randomly-selected clinics.

Of 132 CT scanners in 126 hospitals, complete data were provided for 63 CT scanners (48%) of 57 hospitals. 61 hospitals reported only scanning protocols and the number of patients during the month surveyed, so scanning protocols and the number of the patients undergoing CT examinations for 124 of 132 CT scanners (94%) were obtained. Of 14 clinics (14 CT scanners) to which the questionnaire was sent, complete data were provided by six clinics.

Complete data for individual patients was collected for 19,013 patients scanned by 69 CT scanners.

### • Numbers of patients, CT examinations, and scanning

**Figure 2:**
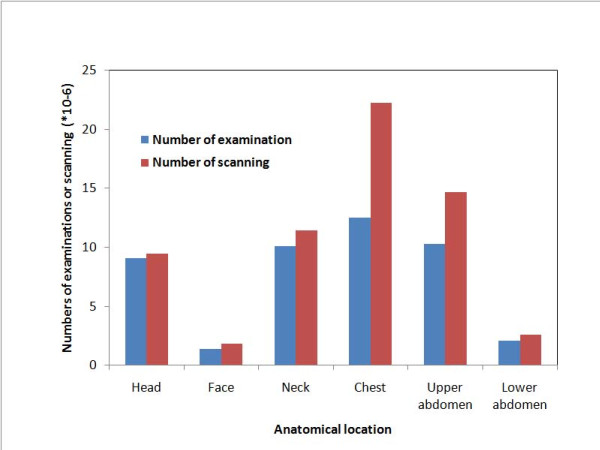
**Estimated annual numbers of CT examinations and scannings in Japan**. The estimated annual number of patients who underwent CT in Japan was approximately 29.9 million. In approximately 50% of these patients, more than two anatomical locations were scanned in one CT session, thus the number of CT examinations was about 45.3 million annually. In addition, approximately 37% of the patients underwent more than two scans of the same anatomical location (for instance, unenhanced and enhanced CT), making the number of scans about 62.5 million annually.

The estimated annual number of adult patients who underwent CT in Gunma prefecture during the month surveyed was 471,557 (Table 2). The estimated annual number of patients undergoing CT per 1000 population would be 235.4. In approximately 50% of the patients, more than two anatomical locations were examined in one CT session; *the estimated annual number of examinations in Japan *was 45.4 million (Figure 2; Table 3). In addition, approximately 37% of the patients underwent more than two scans of the same anatomical location (for instance, unenhanced and enhanced CT). CT examinations of the chest were the most frequent (27%), closely followed by examinations of the upper abdomen (23%), the neck (22%) and head (20%), with examinations of the lower abdomen (5%) and face (3%) being much less frequent. The number of CT examinations for men were larger than those for women.

**Table 3:** Estimated annual number of CT examinations and collective dose in Japan.

CT examination		Annual number of CT examination (*10^6^)	Annual collective dose (*10^3 ^Sv person)
**Head**	Total	9.1	23.6
	Men	4.6	12.1
	Women	4.5	11.5
**Face**	Total	1.4	2.8
	Men	0.7	1.5
	Women	0.7	1.3
**Neck**	Total	10.1	26.5
	Men	5.6	14.8
	Women	4.5	11.7
**Chest**	Total	12.5	120.0
	Men	7.0	67.8
	Women	5.5	52.2
**Upper abdomen**	Total	10.3	83.1
	Men	5.7	47.1
	Women	4.6	36.0
**Lower abdomen**	Total	2.1	21.3
	Men	1.1	12.0
	Women	1.0	9.3

**Total**	Total	45.4	277.4
	Men	24.7	155.3
	Women	20.8	122.1

From these data of Gunma prefecture, an estimated 29.9 million patients are scanned annually in Japan. *The estimated annual number of scans in Japan *was approximately 62.5 million (Figure 2). The *annual number of pediatric patients undergoing CT in Japan *was estimated as 1.43 million. The age and sex distribution for each anatomical location scanned were summarized in Tables 4, 5.

**Table 4:** Estimated annual number of CT examinations for men (a) and for women (b) in Japan for each age range and anatomical locations scanned (*10^6^).

	Age range (yo)								
**CT examination**	**0-19**	**20-29**	**30-39**	**40-49**	**50-59**	**60-69**	**70-79**	**80-**	**Total**

**Head**	0.43	0.17	0.24	0.36	0.53	0.89	1.11	0.89	4.62
**Face**	0.12	0.06	0.06	0.06	0.10	0.14	0.08	0.07	0.69
**Neck**	0.17	0.10	0.28	0.36	0.86	1.66	1.53	0.64	5.60
**Chest**	0.09	0.10	0.25	0.43	0.91	1.86	2.10	1.26	7.00
**Upper abdomen**	0.10	0.09	0.21	0.36	0.74	1.58	1.67	0.94	5.69
**Lower abdomen**	0.02	0.02	0.04	0.07	0.13	0.31	0.32	0.18	1.09

**Total**	0.93	0.54	1.08	1.64	3.27	6.44	6.81	3.98	24.69

**Table 5:** Estimated annual number of CT examinations for men (a) and for women (b) in Japan for each age range and anatomical locations scanned (*10^6^).

	Age range (yo)								
**CT examination**	**0-19**	**20-29**	**30-39**	**40-49**	**50-59**	**60-69**	**70-79**	**80-**	**Total**

**Head**	0.10	0.17	0.23	0.29	0.50	0.73	1.14	1.34	4.50
**Face**	0.11	0.04	0.04	0.05	0.09	0.13	0.16	0.08	0.70
**Neck**	0.00	0.08	0.27	0.59	0.97	1.14	0.96	0.50	4.51
**Chest**	0.09	0.06	0.22	0.42	0.90	1.21	1.30	1.30	5.50
**Upper abdomen**	0.00	0.09	0.22	0.39	0.76	1.03	1.16	0.94	4.59
**Lower abdomen**	0.00	0.03	0.05	0.09	0.16	0.21	0.24	0.22	1.00

**Total**	0.30	0.47	1.03	1.83	3.38	4.45	4.96	4.38	20.80

### • The estimated effective dose, and their distribution

**Figure 3:**
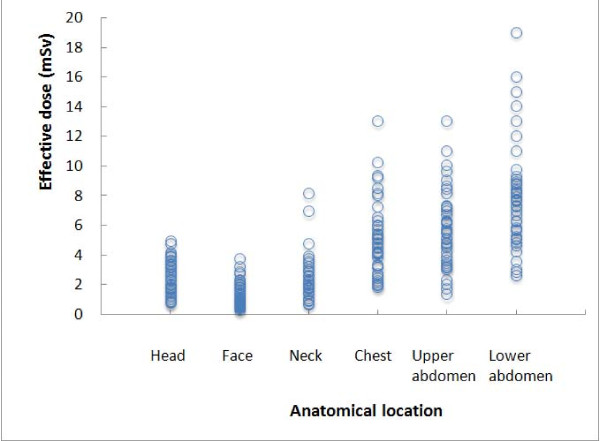
**Distribution of effective dose for one scan for each anatomical location in Gunma prefecture**. The radiation dose of CT examinations varies greatly among institutions.

The mean effective doses from a single scan or examination for each anatomical location were summarized in Table 6 along with comparable data reported from European countries [8-11], and the distributions of the effective dose for one scan were shown in Figure 3. A large variation was observed in the effective dose of one scan among institutions (CT units). In particular, in lower abdominal CT scans, the estimated effective dose ranged from 2.6 to 19.0 mSv. However, there were no factors which were significantly correlated with the estimated effective doses.

**Table 6:** Estimated effective doses per scan and examination for each anatomical location in Japan, and comparison with previous studies.

CT examination		Effective dose for one scanning/one examination (mSv)				
	
	Year Study	2008 Current (Japan)	2000 Japan (13)	2002 Germany (15)	2003 UK (16)	2003-2004 Netherlands (14)
**Head**	Scan	2.5	-	2.2	0.8*	1.9
	Examination	2.6	2.4	2.8	1.5	-
**Face**	Scan	1.5	-	0.8	-	-
	Examination	2.0	-	0.8	-	-
**Neck**	Scan	2.3	-	-	-	-
	Examination	2.6	-	-	-	-
**Chest**	Scan	5.4	-	5.5	3.4*	3.8
	Examination	9.6	9.1	5.7	5.8	-
**Upper abdomen**	Scan	5.7	-	5.5	3.8*	7.2
	Examination	8.1	12.9	11.5	5.3	10.2**
**Lower abdomen**	Scan	8.3	-	6.3	-	-
	Examination	10.3	10.5	7.2	-	-

-: no data.

*: Effective doses for one examination were divided by the number of scanning of the routine sequences.

**: Sum of the effective doses of arterial and portal scans.

### • Estimated annual collective dose, and annual effective dose per capita in Japan

The estimated annual collective effective dose in Japan was 277.4 *10^3 ^Sv person, and CT examinations of the chest and upper abdomen contributed to approximately 73.2% of the collective dose (Table 3). The annual effective dose from CT examinations per capita in Japan was estimated at 2.20 mSv.

## Discussion

The estimated annual number of the patients undergoing CT examinations in this study (235.4 per 1000 population) was slightly lower than that surveyed in 2000 (290 per 1000 population) [13]. Recent advances in CT technology, which enable us to obtain a wide range of CT images, such as neck through the lower abdomen in one session, may partially explain the decrease in the number of the patients undergoing CT examinations. The data of the previous study [13] was obtained from randomly-selected hospitals around Japan, and each hospital provided basic data for a single given week or a single given day. We collected data for a single month from the institutions in a single prefecture. The differences in data collection methods may be the reason for the slightly different results.

The collective effective dose (277.4 * 10^3 ^Sv person) estimated in the current study was slightly lower than that estimated in 2000 for Japan (295 * 10^3 ^Sv person) [13]. This difference may be partially explained by the decrease in radiation exposure to the upper abdomen: the effective dose for one upper abdominal CT examination (8.1mSv) was 37% lower than that obtained in the previous Japanese study (12.9 mSv) (Table 6) [13], and CT examinations of the upper abdomen were frequent (23%). The advance of multi-detector CT technology may have decreased the radiation dose [14]. In the previous Japanese study [13], the collective effective dose for head CTs was estimated around 38 * 10^3 ^Sv person, which was much higher than our result (23.6 * 10^3 ^Sv person ) in spite of an almost equal effective dose for a single examination (2.4 vs. 2.6 mSv). We suspect this decrease may be due to the recent shift from CT to MRI as the mainstay of radiologic examinations of the head.

Radiation dose also varied among countries [13-16]. Although the reasons for these variations are unclear for us, the average doses in the UK are generally lower not only than those of Japan, but also those of Germany and Netherlands.

### • Uncertainties in the estimation, and limitations of this study

From this study, we learned there were significant difficulties in estimating the radiation exposure from CT examinations. There is a large uncertainty associated with the radiation dose estimates in this study. We would like to discuss the uncertainty in the estimation, and limitations of this study. There are two sources of the uncertainties in the estimation in this study: organ dose estimates, and the sample of patients studied.

The study questionnaire surveyed only the standard CT protocols for each anatomical location, but radiologic technologists usually modify the protocol according to patient body size, affecting the radiation dose. Some CT examinations may be performed for screening purposes, and the screening protocol used in these cases may lead to lower dose values than the standard protocol. In the current study, this possibility was not considered, although screening CT has yet to become popular in Japan.

We collected basic data from hospitals and clinics in a single prefecture to estimate the overall situation in Japan. The age distribution of Gunma prefecture is very similar to that of Japan as a whole, and we suspect that the medical environment in Gunma prefecture reflects that of the Japanese national average (Figure 1; Table 1). However, factors such as economic status and access to hospital care may influence medical care a population receives, including medical radiation exposure. Since the average annual medical cost per population was slightly lower in Gunma prefecture than the national average [17-21], basing our estimates on the number of patients undergoing CT in Gunma prefecture may potentially underestimate the number of patients undergoing CT in Japan as a whole by approximately 10%.

In this study, the data was obtained from half of the hospitals and clinics to which the survey was sent. We are not sure whether or not the radiology departments which provided information differ in numbers and types of CT examinations from those which did not provide information. The data was obtained during June, and multiplied by 12 to obtain the annual estimates. June may not be a typical month regarding CT examinations, but the differences in the number of CT examinations throughout the calendar year in Japan were reported to be less than 2% [13].

Paediatric patients were not included in radiation exposure estimates, since calculating paediatric exposure is problematic. Because of the higher radiation doses to paediatric patients for a given CT [7,22], we suspect the issue of medical radiation exposure to the paediatric patients should be separately discussed. Cardiac CT, which has been recently introduced to clinical practice, was also excluded from our analysis. It has been reported that organ doses to lungs and breast are relatively large in cardiac CT [2,8]. Although the individual radiation exposure is relatively large, we suspect this may not greatly affect the overall results of this study.

Despite these limitations, we believe that the simple approach used in this study to estimate radiation exposure from CT examinations is the best available from current data and technology.

### • A large variation in estimated dose among CT scanners, and the methods reducing radiation exposure

There was a large variation in estimated dose among CT scanners. In particular, for lower abdominal CT, the radiation exposure ranged from 2.6 to 19 mSv (Figure 3). This finding indicated that in some institutions, patients probably receive unnecessary radiation exposure, but in others the radiation exposure may not be sufficient to obtain adequate image quality. However, we have failed to find factors which may contribute to the large variation of radiation exposure. In Japan, there is no official system responsible for quality control of CT examinations and radiation exposure to patients, in spite of very strict governmental control of radioisotope management and radiation exposure to medical professionals. We would like to emphasize the importance of a quality control system for CT image quality and patient radiation exposure. Proper feedback, increased education and application of reference dose levels will be important tools to further reduce such institutional differences [9,14].

Some reliable methods which significantly reduce radiation exposure to patients from CT have been proposed [10]. AEC systems adjust radiation dose according to the patient's attenuation, and may reduce the mean tube current by 20-68% (for instance, 20% for thorax, 38% for abdomen, and 32% for abdomen-pelvis) [11,23,24]. This system is installed in recent modern CT scanners, and usually sustains image quality without an increase of noise. In this study, less than half of the CT scanners surveyed had AEC system.

Of course, the most effective way to reduce the radiation exposure to the population from CT is to avoid unnecessary CT examinations. There is a recent trend for diagnostic imaging to be performed as a precaution to avoid malpractice suits (defensive medicine) [25,26]. In addition, we suspect a tendency for Japanese clinicians to rely heavily on imaging examinations because of their extremely heavy workload due to the recent critical shortage of physicians. Patients also seek perfect medicine, and request imaging examinations themselves, which are provided at a relatively modest price in Japan [26,27]. Physicians should carefully consider the risks and benefits before ordering CT.

## Conclusions

The annual number of adult patients undergoing CT examinations in Japan was estimated at 29.9 million, and that of paediatric patients at 1.43 million. The estimated annual collective effective dose in Japan was 277.4*10^3 ^Sv person. The CT examinations of the chest and upper abdomen predominantly contribute to 73.2% of the collective dose. There was a very large variation in radiation exposure from CT among hospitals surveyed.

## Competing interests

The authors declare that they have no competing interests.

## Authors' contributions

Conception and design: YT & KE

Acquisition of data and analysis: YT, HT, HO & KE

Interpretation of data: YT, ATT & KE

All authors read and approved the final manuscript.

## Pre-publication history

The pre-publication history for this paper can be accessed here:

http://www.biomedcentral.com/1471-2342/10/24/prepub
